# Biomechanical Measurement of Rabbit Cornea by a Modified Scheimpflug Device

**DOI:** 10.1155/2016/8271762

**Published:** 2016-07-03

**Authors:** Bo Zhang, Jianjun Gu, Xiaoxiao Zhang, Bin Yang, Zheng Wang, Danying Zheng

**Affiliations:** ^1^State Key Lab of Ophthalmology, Zhongshan Ophthalmic Center, Sun Yat-Sen University, Guangzhou 510060, China; ^2^Guangzhou Aier Eye Hospital, Guangzhou 510288, China

## Abstract

*Purpose*. To explore the probability and variation in biomechanical measurements of rabbit cornea by a modified Scheimpflug device.* Methods*. A modified Scheimpflug device was developed by imaging anterior segment of the model imitating the intact eye at various posterior pressures. The eight isolated rabbit corneas were mounted on the Barron artificial chamber and images of the anterior segment were taken at posterior pressures of 15, 30, 45, 60, and 75 mmHg by the device. The repeatability and reliability of the parameters including CCT, ACD, ACV, and CV were evaluated at each posterior pressure. All the variations of the parameters at the different posterior pressures were calculated.* Results*. All parameters showed good intraobserver reliability (Cronbach's alpha; intraclass correlation coefficient, *α*, ICC > 0.96) and repeatability in the modified Scheimpflug device. With the increase of posterior pressures, the ratio of CCT decreased linearly and the bulk modulus gradually reduced to a platform. The increase of ACD was almost linear with the posterior pressures elevated.* Conclusions*. The modified Scheimpflug device was a valuable tool to investigate the biomechanics of the cornea. The posterior pressure 15–75 mmHg range produced small viscoelastic deformations and nearly linear pressure-deformation response in the rabbit cornea.

## 1. Introduction

A comprehensive understanding of biomechanical properties of the cornea is crucial for a wide range of clinical applications such as keratoconus, tonometry, and refractive surgeries [1–3]. Corneal biomechanics involves the complex interaction between its lamellar collagen structure and the charged, hydrated proteoglycan gel. Many studies have indicated that the stress-strain response in mammalian corneas is typical for anisotropic collagenous tissues [4–6]. Strip extensometry and inflation tests were still the two important methods in biomechanical studies. The former was relatively simple and had low cost but involved three inherent deficiencies and had less reliability: a strip specimen is originally part of the cornea, its curvature flattens, and the back analysis usually considers the effect of the central cornea thickness and ignores the fact of the natural increase in cornea thickness away from the center [7]. The inflation tests were executed by altering the intraocular pressure (IOP) and measuring the apex displacement or central corneal tangential elongation on the intact eyes or corneal rigs, and then biomechanical parameters were calculated by mathematical analysis. Tissue markers on the epithelial and endothelial surfaces such as mercury droplets, adhesive tape marker, or dark and light contrasting regions of the cornea provided by graphite powder combined with digital cameras were usually needed in the inflation tests; however, these methods could disturb the cornea and had limited metrical parameters [8–10].

Pentacam (Oculus, Germany) using Scheimpflug photography as a basis has become a popular clinical device for measuring and characterizing the anterior segment. Many studies have indicated good repeatability and reproducibility in the measurements of topographic corneal thickness, anterior and posterior corneal curvatures, anterior chamber depth, and angle and corneal aberrations [11–19]. With the update of the software version, it also provides parameters such as corneal volume and keratometric power difference, which offer new and technical analyses of the cornea [20, 21]. At present, Pentacam has been used in the range of study types including chronic applications over longer periods as well as dose range studies investigating a no-effect level with respect to lens changes in animal models. Rats, rabbits, dogs, cats, and monkeys all have ocular dimensions, which allow recording of optical sections of their eyes by Pentacam in a reproducible way [22]. The use of animal corneas as approximate models to human corneas in mechanical property characterization studies had been necessary because of the difficulties in obtaining enough human donor corneas.

In this study, we investigated the biomechanics of intact corneas of rabbits subjected to altering of posterior inflation pressures by a modified Scheimpflug device and evaluated the probability of this method.

## 2. Materials and Methods

### 2.1. Specimen Preparation

Eight fresh rabbit eyes from New Zealand white rabbits (2-3 Kg, female) were obtained. A central disk including the corneal button and a 3 mm scleral ring was removed with a pair of curved scissors and this was followed by the removal of the iris, lens, and ciliary body under microscope within 1 hour of eye enucleation. Prior to specimen preparation the central corneal thickness was measured using a pachymeter (DGH1000, DGH Technologies, Exton, PA). The average value and standard deviation were 397.3 ± 13.7 *μ*m. The epithelium of the cornea was kept complete to lessen the effect of hydration in the procedure.

### 2.2. Inflation Tests

The corneas were mounted onto the artificial pressure chamber (K20-2126, Katena, USA). The pressure chamber was filled with saline solution and connected to a small reservoir, whose vertical movement represented the change of intraocular pressure. The actual pressure in the chamber was measured using a differential pressure transducer (YP101, Xinhang Co., China) and the measurements were recorded automatically in Bio-Signal Acquisition System (Chengdu TME Technology Co., China). The whole apparatus was fixed by two clamps between the two pillars of the Pentacam and moved vertically to attain the appropriate position. The maximum pressure applied was 75 mmHg, which was well above the normal physiological level. Three cycles of loading and unloading up to 75 mmHg at intervals of 5 minutes were applied to recondition and stabilize its behavior of the cornea.

### 2.3. Experimental Protocol

The posterior inflation pressure was varied stepwise from 15 mmHg to 75 mmHg and the images were taken at 15, 30, 45, 60, and 75 mmHg. The pressure was always increased with the speed of 15 mmHg/min. All images were taken in 5 minutes after the step increase in pressure to allow enough time for the equilibration of cornea creep. The surface of cornea was drenched using the solution (Dextran40 Sodium Chloride) in 10 seconds before taking images. The examiner adjusted the joystick until perfect alignment was shown, and then, the system automatically took images of the cornea within 2-second period. Only those scans that registered as “OK” were included. Three consecutive scans were captured for intraobserver reliability analysis. The same procedures were repeated on a different pressure level.

### 2.4. Statistical Analysis

Statistical analysis was performed using SPSS software (version 19.0, SPSS, Inc., Chicago, IL, USA). The intraobserver reliability of all parameters was tested using Cronbach's alpha test (*α*) and the intraclass correlation coefficient (ICC). The intraobserver repeatability for each corneal parameter was assessed by the statistical parameters: the within-subject standard deviation, intraobserver precision, and intraobserver repeatability as previously reported [14]. All the data were tested for normality using the Kolmogorov-Smirnov test. The level of significance was set at 0.05.

## 3. Results

### 3.1. Intraobserver Repeatability and Reliability of the Measurements for the Parameters

Like human screening by Pentacam, most parameters such as corneal curvatures, corneal thickness (CCT), anterior chamber depth (ACD), corneal elevations, anterior chamber volume (ACV), and corneal volume (CV) were acquired by the modified Scheimpflug device. With the increase of the inflation pressure, all parameters varied without the loss of high quality of the images, even in high pressure of 75 mmHg.

The modified Scheimpflug device was shown to be highly reliable in the measurements of the parameters such as central corneal thickness, anterior chamber depth, anterior chamber, and corneal volumes (*α* > 0.98; ICCs > 0.96). With the varying of posterior pressure, the reliabilities of the measurements of the parameters did not change significantly (*p* > 0.05, Table 1). High repeatabilities of the measurements of the parameters were also shown and did not alter significantly with the increase of posterior pressures (*p* > 0.05).

### 3.2. The Measurements of Corneal Thickness

**Figure 1:**
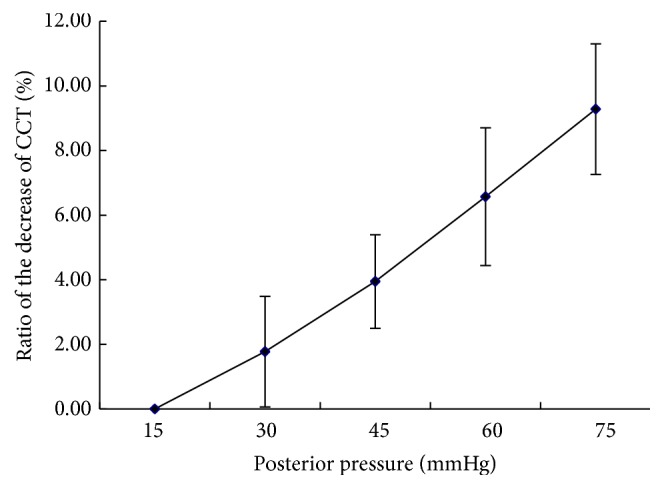
The change of central corneal thickness (CCT) of rabbit eyes with the increase of posterior pressure. The ratios of the decrease of CCT were calculated as (CCT_15 mmHg_ − CCT_posterior  pressure_)/CCT_15 mmHg_, where CCT_15 mmHg_ was viewed as the initial thickness of the cornea. The ratios were shown linearly with the increase of posterior pressure (*R* ^2^ = 0.9928).

The corneal thickness at posterior pressure of 15 mmHg which is appropriately consistent with normal intraocular pressure of rabbit was viewed as the initial number of CCT. With a rise in posterior pressure, the average corneal thickness diminished. The changes of CCT to the primary corneal thickness at pressures were calculated as (CCT_15 mmHg_ − CCT_posterior  pressure_)/CCT_15 mmHg_, and the ratios were shown linearly with the increase of posterior pressure (Figure 1).

### 3.3. Corneal Volume Measurements and Modulus of Volumetric Strain

**Figure 2:**
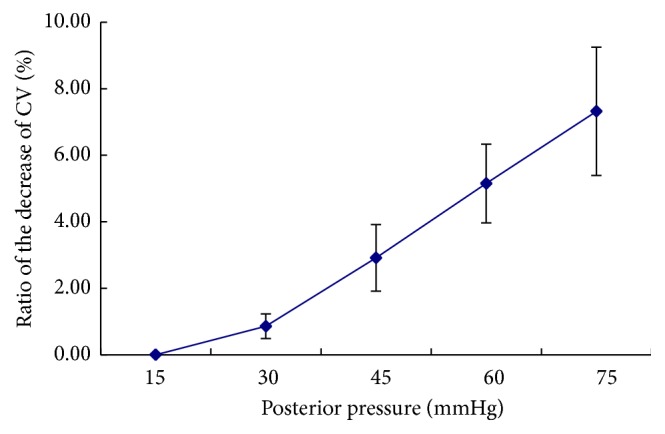
The change of rabbit corneal volume (CV) with the increase of posterior pressure. The ratios of the decrease of CV were calculated as (CV_15 mmHg_ − CV_posterior  pressure_)/CV_15 mmHg_. The ratios were shown linearly with the increase of posterior pressure (*R* ^2^ = 0.98).

**Figure 3:**
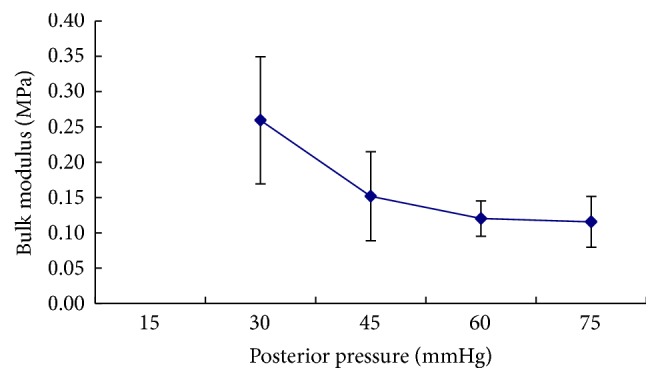
The change of bulk modulus of rabbit corneas with the increase of posterior pressure. The cornea at posterior pressure of 15 mmHg was defined as primary condition (*P*_0_). The bulk modulus was calculated as Δ*P*/Δ*V*, where Δ*P* = (*P* − *P*_0_), Δ*V* = (CV_15 mmHg_ − CV_posterior  pressure_)/CV_15 mmHg_.

Pachymetry software in the device can calculate the corneal volume within different range around the central cornea.  Figure 2 shows the corneal volumes within 10 mm circle decrease with the increase of posterior pressures and the ratios of the decrease of the corneal volume are almost linear. In the inflation test, the stress and volumetric strain values derived from the pressure-deformation experimental results could be used to determine the variation of the bulk modulus with applied posterior pressure. If the cornea at posterior pressure of 15 mmHg was defined as normal primary condition, the average bulk modulus was 0.25 megapascals (Mpa) at the posterior pressure of 30 mmHg and decreased to 0.11 Mpa at 75 mmHg. Beyond the pressure 45 mmHg, the modulus was almost attainted at the platform (Figure 3).

### 3.4. The Measurements of Anterior Chamber

**Figure 4:**
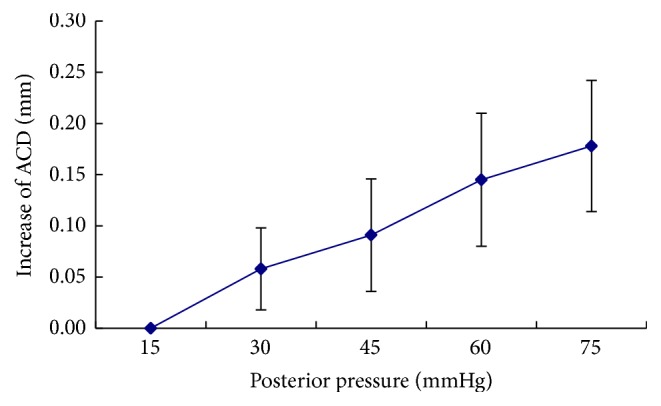
The variation of anterior chamber depth (ACD) of rabbit eyes with the increase of posterior pressure. The increase of ACD was calculated as (ACD_posterior  pressure_ − ACD_15 mmHg_). With the increase of posterior pressures, ACD gradually elevated and the change of ACD was almost linear (*R* ^2^ = 0.9913).

The rabbit corneas were mounted to the artificial chamber for simulating the intact eyeballs. The images of anterior chamber were scanned well by the modified device. With the increase of posterior pressures, both ACV and ACD gradually elevated and the change of ACD was almost linear (Figure 4).

## 4. Discussion

The present study employed a new modified Scheimpflug device and executed the inflation test to examine the rabbit corneal biomechanics. In the experiment, we mounted the rabbit corneas to the artificial chamber. The base of the artificial chamber was modified by black tape to simulate the iris structure. The light reflex reduced greatly and the back surface of the cornea was shown obviously in the scan of the modified device and high quality of the images of anterior segment was acquired. The objective of this study was to evaluate the intraobserver repeatability and reliability of the modified device. In general, a value of 0.70 is considered satisfactory and in clinical applications a value of 0.90 is required. For the ICC, a value above 0.75 indicates good reliability, but most clinical applications require a value of at least 0.90 [13]. In the experiment, the values of ICC and *α* were almost 1.0 and excellent results even higher than those in human measurements as reported were shown in repeatability analysis [13]. There were two reasons to account for the facts: firstly, the device kept stable without any factors affecting the measurement such as eye movement or blink during the scan. Secondly, several studies reported that the creep was small for the normally hydrated and the swollen corneas and lasted between 30 and 70 seconds [23], so the posterior pressures continued for 5 minutes and ensured the equilibration of corneal creep without effects on the measurements of the cornea in the study. Our results indicated that the modified device had good reliability and repeatability in the measurements of the parameters, even still kept stable with the increase of posterior pressures. The modified device should be taking advantage of measuring the corneal biomechanics.

Until now corneal biomechanics has been challenging in the ophthalmic field because of difficulties of in vivo accurate measurements. Although two noncontact tonometers (Ocular Response Analyzer and CorVis ST) are currently commercially available to study corneal biomechanics, there are still obvious drawbacks in the two devices. In the former, whether the corneal resistance factor and corneal hysteresis accurately represent corneal biomechanics is questionable, even some studies have found that there is no direct relationship between the modulus of elasticity and the corneal hysteresis and this suggests that conclusions from studies using the ORA should be considered cautiously [24, 25]. In the latter, the Corvis ST does not provide conventional biomechanical parameters such as the elastic modulus and the stiffness. Furthermore, the area of analysis with the two devices is only limited to approximately the central 3.0 mm of the cornea, which does not represent all of the corneal biomechanics [25, 26]. The inflation test is still a valuable method combined with shell theory to calculate stress, strain, and elastic modulus, which ensures the integrity of the corneal tissue in almost normal physiological condition.

Some studies have proved that corneal hydration affects the properties of corneal biomechanics and corneal thickness rises linearly with the increase of corneal hydration [27]. In the study, we kept the intact epithelium and minimize the evaporation on corneal surface drenched using the solution (Dextran40 Sodium Chloride) and the corneal hydration almost maintained normally in the procedure to minimize its effects on the properties of corneal biomechanics. In biomechanical studies, the corneal strain behaviors and corneal apex displacement were usually investigated. One study found that the human cornea showed a negligible extensibility and the rabbit tissue underwent a 9% strain with a curvilinear relationship between stress and strain under low pressures and the relationship was linear at higher pressures [28]. Our data showed the linear relationship between posterior pressures and ACD increase similar with apex displacement beyond the normal physiologic IOP and they were consistent with previous reports.

In this study, we found that the corneal thickness decreased linearly with increasing intraocular pressure and the corneal thickness decreased by 9.28 ± 2.02% for a pressure increase from 15 mmHg to 75 mmHg, which was in good agreement with the results of Hennighausen et al. [23]. According to the incompressibility assumption, corneal volumetric considerations reveal that the relative change in stromal thickness would have been twice the tangential strain of the stroma and the reduction of the corneal thickness is generally viewed as compensation for the corneal surface increase on inflation test. But the average corneal thickness decreased more than expected theoretically; a small reduction in corneal volume did take place during the pressure increase [29]. The modified Scheimpflug device can provide the data of corneal volume in different range from the apex of the cornea and may benefit investigating the interaction of fluid flows within the stroma and corneal load. In the experiment, our data showed that the corneal volume decreased by approximately 7.32% for a pressure increase from 15 mmHg to 75 mmHg and it further supported the idea that local fluid shifts in the stroma are possible within minutes after load changes.

Young's modulus is a measure of property of elastic materials and it is defined as the ratio between stress and strain. Mammalian corneas demonstrated hyperelastic behavior with an initial low stiffness and a final high stiffness under short-term inflation testing. The stress-strain relationship of the cornea is not linear. Therefore, it is inappropriate to use Young's elastic modulus to describe the nonlinear biomechanical properties of the cornea [30]. In this study, bulk modulus defined as the ratio of the infinitesimal pressure increase to the resulting relative decrease of the volume was introduced to describe the corneal biomechanics. The changes of the corneal bulk modulus were investigated with the posterior pressure increase and we found that the bulk modulus decreased gradually to the platform. It suggested that the cornea was almost viewed as an incompressible soft tissue especially with the stress of more high posterior pressure.

In conclusion, the modified Scheimpflug device is a valuable method to measure the parameters of anterior segment on the inflation tests and has more advantages of the measurements of biomechanics of the cornea. The variations of the corneal biomechanics due to the refractive corneal surgeries or corneal transplantation may be further investigated by this device.

## Figures and Tables

**Table 1:** Summary of intraobserver repeatability and reliability for the parameters.

Parameters	Posterior pressure (mmHg)	Sw	Pr	Rep	C alpha	ICC
CCT	15	0.972	1.904	2.691	1.000	1.000
	30	1.028	2.014	2.847	1.000	1.000
	45	1.374	2.694	3.807	1.000	0.999
	60	1.460	2.863	4.046	1.000	0.999
	75	1.713	3.357	4.744	1.000	0.999

ACD	15	0.032	0.062	0.088	0.998	0.994
	30	0.032	0.062	0.088	0.998	0.995
	45	0.032	0.062	0.088	0.998	0.996
	60	0.032	0.062	0.088	0.999	0.997
	75	0.032	0.062	0.088	0.999	0.998

ACV	15	2.402	4.709	6.655	0.998	0.993
	30	5.911	11.586	16.374	0.988	0.967
	45	2.448	4.799	6.782	0.998	0.995
	60	3.141	6.157	8.701	0.997	0.994
	75	6.011	11.782	16.651	0.994	0.976

CV	15	0.138	0.270	0.382	1.000	0.999
	30	0.118	0.232	0.328	1.000	1.000
	45	0.276	0.540	0.764	0.999	0.997
	60	0.138	0.270	0.382	1.000	0.999
	75	0.443	0.868	1.226	0.997	0.988

Sw: within-subject standard deviation; Pr: precision; Rep: repeatability; ICC: intraclass correlation coefficient; CCT: central cornea thickness; ACV: anterior chamber volume; ACD: anterior chamber depth; CV: corneal volume.
